# Supplementary material for: A Systematic Review of Compensation and Technology-Mediated Strategies to Maintain Older Adults’ Medication Adherence
Source: Int J Environ Res Public Health. 2023 Jan 1;20(1):803. doi: 10.3390/ijerph20010803 (PMC9819645; doi:10.3390/ijerph20010803)
Supplement: Supplementary file 1 [file ijerph-20-00803-s001.zip › ijerph-2078527-supplementary.pdf]

Suplementary Table S1. Summary of characteristics of included studies.

No	Author	Year	Study Design	Country	Strategies/ intervention	Participan ts	Participants Type	duration of interventions	Measurement	Findings
1	Branin et al	2001	Cross-Sectional	United States	Memory strategies	190	Older adults over the age of 64 living independen tly in the community	NE	The 36-Item Short-Form Health Survey (SF-36), he 15-item Geriatric Depression Scale (GDS), The Medication Taking Question- naire, a self-administered instrument	In their medication adherence practices, older persons reported using internal memory strategies more frequently than external memory strategies. The internal memory approach most frequently used was to relate taking medication to one's daily routine. The most popular external memory strategy was using an outdated pillbox.
2	Swanlu nd	2010	Qualitativ e study	United States	Simplification , use of visual and tactile cues, and development of a medication routine, knowledge and education, mental alertness, determinatio	27	community -dwelling adults over age 74	45 to 90 minutes.	Qualitative analysis	The most commonly mentioned effective strategies were procedural simplification, the implementation of a medication routine, the development of tactile and visual cues, and medication awareness and education. These results can be used to assist older persons in developing effective cardiovascular drug management strategies.

					n, caregiver set up					

No	Author	Year	Study Design	Country	Strategies/ intervention	Participan ts	Participants Type	duration of interventions	Measurement	Findings
3	Harbig et al	2012	RCT	Denmar k	electronic reminder system called Telesvar (TS) and pill count	168	home-living elderly patients, taking more than four drugs per a day	1 year	the adherence measured by the electronic device with the actual adherence measured by the pill count	The electronic reminder method showed a 79% adherence rate, and pill count showed a 92% adherence rate. For the purpose of revealing adherence behavior patterns, electronic monitoring devices may be helpful. When forgetfulness is the main source of the non-adherence issue, electronic reminders may also be helpful.

4	Boron et.al	2013	Cross-Sectional	United States	Pill caddy, association, external physical reminder, location, mental planning, physical pain, and visibility.	354	older adults in the Atlanta Metropolitan Area	NE	The questionnaire (available from the authors) contained four sections: (1) General background, health, and medical product usage questions, (2) Strategies used to remember to take medical products, (3) General medication behavior questions, and (4) Attitudes about remembering medications questions.	The location strategy was the most frequently endorsed, with 61% responding that they used this strategy. The visibility and association strategies were endorsed by 43% and 40% of respondents, respectively. Use of a pill caddy was endorsed by 36% of the sample. Mental planning was used by 23% of respondents. Only 11% and 7% reported use of physical pain and external reminders, respectively.
No	Author	Year	Study Design	Country	Strategies/ intervention	Participants	Participants Type	duration of interventions	Measurement	Findings

No	Author	Year	Study Design	Country	Strategies/ intervention	Participants	Participants Type	duration of interventions	Measurement	Findings
5	Kinyua et. al	2013	Cross-Sectional	Kenya	Send SMS, Alarm use, Alarm purpose	500	patients receiving Highly Active Anti-Retroviral Therapy (HAART) at the Kenyatta National Hospital Comprehensive Care Clinic in Nairobi between May and July, 2011.	NE	questionnaires in English or Kiswahili	The majority of HIV-infected individuals interviewed agreed that using cell phones to control their HIV infection was a nice choice. A high proportion said they needed mobile phone connectivity for medical assistance and guidance on factors that affect medication adherence, while only 3% said they needed it as a reminder to take their medications. For convenience and confidentiality, the majority favored phoning the healthcare provider with their own phones, with only 0.4% preferring to be phoned or texted by the health care provider.

6	Wang et al	2013	RCT	Taiwan	A volunteer coaching program (a coach and reminders by well-trained volunteers, as well as three home visits and five telephone calls over a two-month period)	62 (30 elderly participants were assigned to routine care plus volunteer coaching, with the remaining 32 assigned to routine care only.	community-dwelling older adults who were 65 or older and resided in a remote rural area of southern Taiwan	two months	22-item researcher developed structured-questionnaire (KAB-MS), based on the Medication Safety Guide.	The volunteer coaching group improved overall understanding of medication safety, but there was no change in attitude at the end of the two-month study period. Furthermore, when compared to the normal care group, the group displayed three improved medication safety behaviors. Community health practitioners who want to improve the medication safety of chronically ill elders can use the volunteer coaching program and instructions with pictorial aids as a reference.
No	Author	Year	Study Design	Country	Strategies/ intervention	Participants	Participants Type	duration of interventions	Measurement	Findings

7	Brooks et.al	2014	Mixed-Method	United States	keeping the medication in a usual location, integrating medication use with a daily routine, taking the medication at a specific time, taking the medication with other medications, using the medication only when needed, and using other reminders	328	English-speaking and Spanish-speaking adults ages 60 years and older with moder- ate or severe asthma	3 months	the Medication Adherence Rating Scale (MARS), open-ended questioning, Brief Illness Perceptions Questionnaire, The Beliefs about Medicines Questionnaire, the Generalized Anxiety Disorder 7 questionnaire, the Short Test of Functional Health Literacy in Adults (S-TOFHLA)	The good adherence rate was greater among individuals who kept their ICS medication in the bathroom or integrated its use into a daily routine.

8	Vollmer et al.	2014	RCT	the Kaiser Permanente (KP) health plan-Northwest (KPNW), Hawaii (KPH), and Georgia (KPG)-United States	either usual care (UC) or 1 of 2 interventions in the form of interactive voice recognition calls—regular (IVR) or enhanced (IVR+)	21752	participants who were 40 years or older, had diabetes mellitus or atherosclerotic cardiovascular disease, and were suboptimally adherent.	1 year	a modified version of the Proportion of Days Covered (PDC), Other EMR-Based Data	Compared to UC, both therapies significantly improved adherence to statins and ACEIs/ARBs (angiotensin-converting enzyme inhibitors/angiotensin receptor blockers). Additionally, IVR+ participants had considerably stronger ACEI/ARB adherence than IVR participants. IVR+ individuals exhibited significantly reduced low-density lipoprotein (LDL) levels at follow-up compared to UC participants among statin users.
No	Author	Year	Study Design	Country	Strategies/ intervention	Participants	Participants Type	duration of interventions	Measurement	Findings

No	Author	Year	Study Design	Country	Strategies/ intervention	Participants	Participants Type	duration of interventions	Measurement	Findings
9	Goldstein et.al	2014	RCT	Ohio, United States	Patients were randomized to one of four groups: smartphone silent, smartphone reminding, pillbox silent, pillbox reminding.	60	older adults, patient with heart failure	10 months	A daily adherence value was calculated by dividing the number of recorded medication-taking events by the number of scheduled medication-taking events for all four medications. Therefore, medication adherence was measured by pillbox bin openings for people with the telehealth intervention and electronic self-report for people with the m-health intervention.	people with the telehealth device adhered 80% of the time and people with the smartphone adhered 76% of the time. medication reminding via an alarmed electronic pillbox (telehealth) or a smartphone app (m-health) produced similar medication adherence

10	Ligons et.al	2014	Quasi-experiment	Pittsburgh, United States	EMMA® (Electronic Medication Management Assistant), EMMA® delivers medications from single-dose blister cards, according to schedules programmed remotely by prescribing pharmacies.	19	The subject pool was primarily white, female, 80+ and in possession of above average education	1 months	Video coding allowed for quantification of usability errors observed during the testing sessions, cognitive status was assessed using the Mini Mental State Exam (MMSE®), Functional status was assessed using the Lawton Instrumental Activities of Daily Living (IADL) questionnaire, subjective usability was assessed by completing the System Usability Scale (SUS)	By integrating reminder systems with telemedical monitoring and control capabilities, drug delivery systems like EMMA® have the potential to enhance medication management.
No	Author	Year	Study Design	Country	Strategies/ intervention	Participants	Participants Type	duration of interventions	Measurement	Findings

11	Mira et.al	2014	RCT	Spain	An app for managing medications on tablets (called ALICE). In order to allow for the customization of prescriptions and medical advice, ALICE was developed for Android and iOS. In addition to alerts and multiple reminders for each alert, it displays images of each medication, both the container and the medication itself.	99 (48 and 51 in the control and experimental groups)	multimorbid patients taking multiple medications, older than 65 years	3 months	rate of missed doses and medication errors reported by patients, scores from the 4-item Morisky Medication Adherence Scale (MMAS-4), level of independence, self-perceived health status, and biochemical test results	Patients in the experimental group scored higher on the MMAS-4 and reported missing fewer doses of their medications. Only patients with an initial greater prevalence of medication mistakes have seen a significant reduction due to ALICE. The ALICE app boosts perceived independence in handling medications, increases adherence, and aids in lowering rates of forgetting and prescription errors.

No	Author	Year	Study Design	Country	Strategies/ intervention	Participan ts	Participants Type	duration of interventions	Measurement	Findings
12	Grindro d et. al	2014	Mixed-Method	Canada	An iPad loaded with four medication management applications: MyMedRec, DrugHub, Pillboxie, and PocketPharm acist	35	Participants who were at least 50 years old, could read and speak English, and were taking a chronic drug	2 hours	the 10 item System Usability Scale (SUS) and visual analog scale	After some time and instruction, the majority of participants felt comfortable using the applications, but their initial interactions with them left them frustrated. Although it included a medication interaction function that was one of the users' favorite features, participants preferred MyMedRec and DrugHub for their simple, linear interfaces, and it was determined that PocketPharmacist was the least usable of the four applications.
No	Author	Year	Study Design	Country	Strategies/ intervention	Participan ts	Participants Type	duration of interventions	Measurement	Findings

13	Stawarz et al	2016	Survey study	UK and Mexicali, Mexico	everyday routines, physical objects used as visual cues and the location of their medications.	971 women taking oral contracep tion, 88 parents of children who took antibiotics within the past year, and 87 older adults.	older adults with healthcare and social services	NE	The first part included 18 questions that investigated how respond- ents remembered their medications, and how often and why they forgot. The second part included the standardized 16-item self-report Prospective and Retrospective Memory Questionnaire (PRMQ)	contextual cues, routine events, locations, and meaningful objects is a common and often effective strategy; combinations of two or more types of cues are more effective than relying on a single cue.
14	Mertens et. al	2016	Quasi-experiment	Germany	The mobile application Medication Plan, installed on an Apple iPad	24	Patients with coronary heart disease and no prior knowledge of tablet computers	an initial phase without assistive systems (between 3 and 6 months in line with standard rehabilitation treatment after an inpatient hospital stay), an interventiona	Subjective adherence was determined by the A14-scale, Technical knowledge and experience were assessed using an adapted version of the computer literacy scale (CLS),	Logging data showed a significantly stronger adherence for the medication app than the paper system for both blood pressure recordings ($P < 0.001$) and medication intake ($P = 0.033$). A mobile app for medication adherence increased objectively and subjectively measured adherence in elderly users undergoing rehabilitation.

								l phase (28 days of using the app system), and a comparative phase (28 days of using a paper diary)		
No	Author	Year	Study Design	Country	Strategies/ intervention	Participants	Participants Type	duration of interventions	Measurement	Findings
15	Dasgupta et. al	2016	Cross-Sectional	United States	A comprehensive system that focuses on quality of life, eSeniorCare, includes a tablet application and a web portal.	16	a local independent living facility catering to a low-income senior population	3 months	edication Usage Logs (MUL), Medication Adherence Questionnaire (MAQ), Final Feedback Questionnaire (FFQ), A 12-item health survey provided by Quality Metric to measure the general health	Elderly may successfully track their medications with the help of eSeniorCare, which offers an intersection of technology and relationship-building.
No	Author	Year	Study Design	Country	Strategies/ intervention	Participants	Participants Type	duration of interventions	Measurement	Findings

16	Varleta et al.	2017	RCT	Chile	mobile phone short message system (SMS) text messaging, patients were randomly assigned to receive or not receive SMS related to ADA and healthy lifestyle.	314	patients with hypertension with <6 months of antihypertensive treatment from the Preventive Health Program of 12 different primary care centers	6 months	self-reported four-item scale Morisky-Green-Levine questionnaire	By contrast, adherence improved significantly in the SMS text message group from 49% to 62.3%.
17	Tofighi et al.	2017	prospective cohort study	New York, United States	text message reminder	93	office-based buprenorphine treatment (OBOT) patients	6 months	The initial 16-item feasibility survey was based on the Technology Acceptance Model	All OBOT patients should receive reminders, according to the respondents, who also said that reminders helped patients keep their planned appointments (97% of the time). The majority of participants stated that the TM reminders helped them keep appointments (95%), that all clinic patients should be able to receive them (95%), and that they preferred getting solely TM reminders over telephone reminders (95%).

No	Author	Year	Study Design	Country	Strategies/ intervention	Participants	Participants Type	duration of interventions	Measurement	Findings
18	Pagan et. al	2018	Mixed-Method	United States	mobile phone text message intervention	21	individuals aged 50-68 years, who self-identified as African American, HIV+, currently on ART	8 weeks	three-item self-report measure examining ART use in the prior 4 days, Adherence self-efficacy was assessed using the HIV Adherence Self-Efficacy Scale (HIV-ASES), a 12-item self-report scale of patients' confidence in carrying out behaviors related to treatment adherence, including taking medications in the face of barriers, qualitative analysis	Participants reported statistically significant increases in medication adherence after 8 weeks, but not in their affect or self-efficacy scores. Qualitative findings highlight the intervention's potential for psychological support, adherence issues, and ideas for development. The study suggests that an ART adherence support text message intervention may be feasible for older African Americans living with HIV.
No	Author	Year	Study Design	Country	Strategies/ intervention	Participants	Participants Type	duration of interventions	Measurement	Findings

19	Adler et al.	2019	prospective cohort study	Ghana	interventions where community-based cardiovascular disease (CVD) nurses were trained by FHI 360	1339	All adult hypertensive community members, except pregnant women, were eligible for inclusion in the study.	1 year	The main outcomes of interest were hypertension control (<140/90 mm Hg) and changes in SBP and DBP	72% of patients had controlled hypertension after receiving treatment for a year. Both the systolic and diastolic blood pressures were decreased by 12.2 and 7.5 mm Hg, respectively. Education, advanced age, having controlled hypertension at enrolment, and the date of enrolment were all linked with finishing the program for 12 months. Most of the patients that persisted in the program were receiving therapy, with two thirds consuming two or more medication.
No	Author	Year	Study Design	Country	Strategies/ intervention	Participants	Participants Type	duration of interventions	Measurement	Findings

20	Shen et. al	2019	RCT	Beijing, China	low-cost reminder package (LCRP) combined with case-based health education or usual care	518	Essential hypertensive patients who volunteered to participate, aged above 18, diagnosed according to ICD-9 codes 401–401.9, and currently taking at least one kind of antihypertensive medications.	6 months	two questionnaires on baseline and six-month follow-up	Medication adherence (MA) scores between the intervention group and the control group did not differ substantially at the beginning of the study. According to the outcomes of multilevel modeling, the intervention group's MA scores increased more than the control group's. Combining LCRP with case-based health education could greatly enhance the MA of hypertension patients.
21	HYH Siu et al	2019	RCT	Ontario, Canada	the eDosette intervention	57	community-dwelling older adults	4 weeks	the participant’s observed medication adherence rate was calculated by dividing the total medication doses taken correctly (numerator) by the total doses in the study (denominator).	The mean overall adherence rate was 82% (range 49%-100%). The eDosette intervention can be feasibly implemented into the primary care setting to provide Primary Care Teams with previously unavailable information on when an older adult takes their medications

No	Author	Year	Study Design	Country	Strategies/ intervention	Participants	Participants Type	duration of interventions	Measurement	Findings
22	Park et. al	2020	Qualitative study	Northern California, United States	text messaging and mobile phone applications	28	veterans and non-veterans with a history of coronary heart disease and antiplatelet medication use	1-1.5 hour (3 session @ 2 weeks)	Qualitative analysis	Older adults are engaged and proficient mobile technology users, perceiving TM and mobile phone apps as helpful tools in their medication adherence.
23	Raj et al.	2020	RCT	India	the usual standard of care or the intervention that comprises of systematic education, patient diary to mark daily medicine intake and periodic telephone reminders.	50 (25 control and 25 intervention)	older (age >60 years) inpatients of general medicine wards diagnosed with select non-communicable diseases, such as diabetes, hypertension, dyslipidemia and coronary	6 months	laboratory parameters, clinical signs and symptoms, reported pill count (RPC), prescription changes, barriers to adherence, and KAP	The reported pill count (RPC) change within groups and between groups at the third and sixth months served as the main endpoints. At three months, the RPC in the control and intervention arms were 78.20% and 91.88%, respectively, while at six months, they were 68.64% and 83.08%. Similar to this, there was a statistically significant change in RPC in the intervention arm between baseline and the third month and between baseline and the sixth month.

							artery disease			
No	Author	Year	Study Design	Country	Strategies/ intervention	Participan ts	Participants Type	duration of interventions	Measurement	Findings

24	Vieira et.al	2020	Mixed-Method	Brazil	a monthly electronic medication organizer device equipped with an alarm clock, called Electronic System for Personal and Controlled Use of Medications (Supermed)	32	older adult patients with diagnosis of hypertension, who were recruited at a Primary Care Unit in Brazil.	Pre-intervention period: five meetings, Intervention day : one meeting , Post-intervention period: five meetings (one meeting per month)	Morisky Medication Adherence Scale	In terms of medication adherence, 81.2% of patients showed "lowered adherence" before the intervention and 96.9% showed "greater adherence" after it. Between intervention day and post-intervention, the mean systolic and diastolic blood pressure differences were 18.5 mmHg and 4.3 mmHg, respectively, and between pre-intervention and post-intervention, the differences were 21.6 mmHg and 4.7 mmHg, respectively. In a cohort of hypertensive older adults, the use of Supermed dramatically increased self-reported medication adherence and blood pressure control.
No	Author	Year	Study Design	Country	Strategies/ intervention	Participants	Participants Type	duration of interventions	Measurement	Findings

25	Patel et al	2020	mixed methods study	Canada	21 electronic medication adherence products (GMSa Med-e-lert automatic pill dispenser, LiveFine automatic pill dispenser and reminder, MedReady 1700 automated medication dispenser, MedSmart med-reminder and dispensing system, e-pill MedTime Station automatic pill dispenser with tipper, TimerCap travel size, TimerCap universal size, Jones medication adherence	39	older adults taking one or more prescription or nonprescription medications regularly, caregivers of older adults, and health care providers.	NE	the system usability scale (SUS) and NASA-task load index (NASA-TLX)	Electronic medication adherence products required a mean of 12.7 steps (range 5-20) for the appropriate use and took, on average, 15.19 min to complete the setup tasks (range 1-56). Higher usability scores indicate that an older adult, caregiver, or health care provider is more likely to be able to use a product appropriately, potentially decrease medication error rates, and improve adherence

					system 14-unit card, Reizen vibrating pill box, VitaCarry advanced pill case, Nishiki round pill box with alarm, MedGlider system 1 with talking reminder, Patterson medical tabtime super 8, 100-Hour pill reminder, Med-Q smart pill box, e-pill MedGlider home medication management system, MedCentre system, eNNOVEA Weekly Planner With Advanced Auto					

No	Author	Year	Study Design	Country	Strategies/ intervention	Participants	Participants Type	duration of interventions	Measurement	Findings
					Reminder, e-pill Multi-alarm pocket XL, 6 Grid pill storage case with alarm, Itzbeen pocket doctor)					

26	Alasfour et.al	2020	RCT	Saudi Arabia	"My Dear Knee" application, Arabic smartphone application on adherence to home exercise programs (HEPs)	40	women aged >50 years with knee osteoarthritis	6 weeks	Self-reported exercise adherence (The adherence rate was obtained as a percentage of exercises completed out of the total number of prescribed exercises)	By the end of week 6, the app group had reported improved adherence to HEPs and a significant decrease in pain.
27	Mao et.al	2022	Cross-Sectional	China	the intelligent medication administration system and The WeChat Program Smart Drug Box	354	aged over 60 years, diagnosed with at least one chronic disease, prescribed at least two medications	two weeks	A self-made questionnaire	Six options for the final Kano component, which was determined to be indifferent, included having simple and attractive displays, automatically blocking advertisements, a user privacy protection protocol, allowing logged-in users to view personal medical information, keeping track of all prescriptions, and ordering medications through WeChat. Researchers should place more emphasis on enhancing older adults' experiences while facilitating their usage of the intelligent drug administration system, according to older adults'

										individual needs and desires.

RCT: Randomized Controlled Trial; NE: Not Explicitly; SF-36: The 36-Item Short-Form Health Survey; GDS: The 15-item Geriatric Depression Scale; EMMA®: Electronic Medication Management Assistant; MMSE®: the Mini Mental State Exam; IADL: the Lawton Instrumental Activities of Daily Living; SUS: the System Usability Scale; PRMQ: the standardized 16-item self-report Prospective and Retrospective Memory Questionnaire ; SMS: Short message system; RPC :Reported Pill Count; KAP questionnaire : Knowledge, Attitude, and Practices; SBP: Systolic Blood Pressure; DBP: Diastolic Blood Pressure; ACEIs/ARBs : angiotensin-converting enzyme inhibitors/angiotensin receptor blockers.
